# Meeting of the National Association of Dental Faculties

**Published:** 1895-09

**Authors:** 


### Meeting of the National Association of Dental Faculties.

The annual session of this body was held at Asbury Park, August 3rd, 5th and 6th, 1895, Dr. Frank Abbott President. There was quite a large attendance, every college having membership was represented, thirty-three in all; five were admitted to membership, making in all thirty-eight.

Much important business was done, all of which will tend to the elevation of dental education. One of the most important was the increasing the college term to six months; that regulation to take effect with the session of 1896–7. A resolution was passed to make it seven months, but there was a feeling on the part of some that that was a greater step than should be taken at once, though all admitted that seven months was not too long; after thinking over the matter for a time, and out of deference to the views of those who regarded seven months too great a step to take at once, and to secure entire unanimity of action, a reconsideration was proposed and carried; and the six months term was unanimously adopted. This action will certainly result in the greater efficiency of the colleges; it will enable them to bestow more time upon the work of the course and also to somewhat extend their curriculum, if they desire to do so, both of which is in the line of progress.

This Association is exercising greater influence for the elevation of the profession than any other one agency, and especially is this true in respect to the future of dentistry; it does not much affect the present practitioners, only in a reflex way, but it is prophetic of great things for the future.

The officers for the present year are: President, Dr. S. H. Guilford, of Philadelphia, Pa.; Vice-President, Dr. Geo. H. Cushing, of Chicago, Ill.; Secretary, Dr. Louis Ottofy, of Chicago,

Ill.; Treasurer, Dr. H. W. Morgan, of Nashville, Tenn.; Executive Committee, J. Taft, of Cincinnati, Thos. Fillebrown, of Boston, and B. Holly Smith, of Baltimore.; Ad Enterim Committee, T. W. Brophy, of Chicago, Dr. A. O. Hunt, of Iowa City, Iowa, and H. A. Smith, of Cincinnati, Ohio.

From the great interest manifested, and the many important questions that ought to be considered by that body, it is well nigh a certainty that there will be a full, and, it is to be hoped, a unanimous, attendance next year. There are so many subjects of a practical nature that ought to have attention that no member can afford to be absent.

